# Multispectral High Temperature Thermography

**DOI:** 10.3390/s22030742

**Published:** 2022-01-19

**Authors:** Waldemar Wójcik, Vladimir Firago, Andrzej Smolarz, Indira Shedreyeva, Bakhyt Yeraliyeva

**Affiliations:** 1 Department of Electronics and Information Technology, Faculty of Electrical Engineering and Computer Science, Lublin University of Technology, Nadbystrzycka 38d, 20-618 Lublin, Poland; waldemar.wojcik@pollub.pl (W.W.); a.smolarz@pollub.pl (A.S.); 2 Department of Quantum Radiophysics and Optoelectronics, Belarusian State University, 4 Nezavisimosti Avenue, 220030 Minsk, Belarus; 3 Faculty of Information Technology, M.Kh.Dulaty Taraz Regional University, Tole Bi St 40, Taraz 080000, Kazakhstan; indishera@mail.ru (I.S.); b_eral@mail.ru (B.Y.)

**Keywords:** temperature measurement, thermography, pyrometry methods, thermal radiation coefficient, RGB matrices, high-temperature thermal imaging

## Abstract

The paper considers the issues of creating high-temperature digital thermographs based on RGB photodetector arrays. It has been shown that increasing the reliability of temperature measurement of bodies with unknown spectral coefficient of thermal radiation can be ensured by optimal selection of the used spectral range and registration of the observed thermal radiation fields in three spectral ranges. The registration of thermal radiation in four or more spectral ranges was found to be inefficient due to the increasing error in temperature determination. This paper presents a method for forming three overlapping spectral regions in the NIR spectral range, which is based on the use of an external spectral filter and a combination of the spectral characteristics of an RGB photodetector array. It is shown that it is necessary to ensure the stability of the solution of the system of three nonlinear equations with respect to the influence of noise. For this purpose, the use of a priori information about the slope factor of the spectral dependence of the thermal radiation coefficient in the selected spectral range for the controlled bodies is proposed. The theoretical results are confirmed by examples of their application in a thermograph based on an array of CMOS RGB photodetectors.

## 1. Introduction

**Figure 1:**
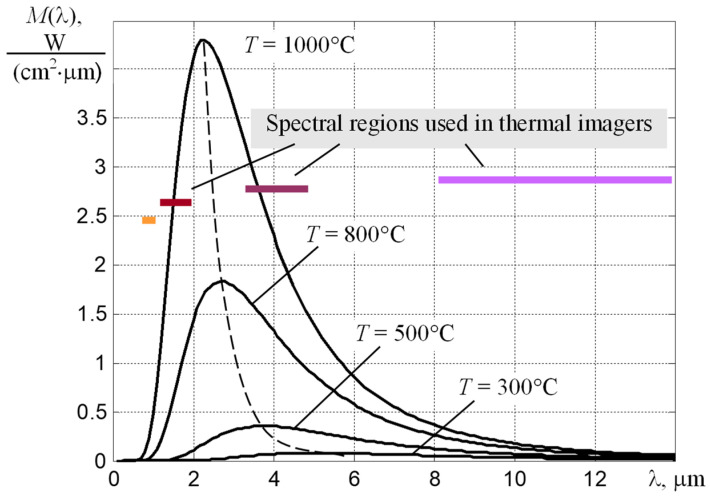
Dependences of the spectral density of the surface luminosity of the absolute black body on the wavelength at different temperatures and spectral regions that are used in modern thermal imagers of the NIR, SWIR, MWIR, and LWIR spectral ranges.

Today’s product quality requirements impose a mandatory control of the conditions under which manufacturing processes are carried out. Process temperature control is one of the main requirements of modern standards. Temperature is a quantitative parameter of the internal energy of bodies, so recording and analysis of temperature fields gives information on the state of objects and the course of various processes [1,2]. The non-contact methods based on the registration of thermal radiation emitted by the surface of the heated product seem to be the most convenient ones for controlling the temperature of complex thermal processes. In many cases, it is effective to use an infrared imaging technique that enables the presentation of a partial image of the radiation temperature field *T_r_* (*x*,*y*) of the supervised body on the monitor screen. Extensive information on its capabilities and application can be found on the websites of leading manufacturers of thermal imaging and thermographic equipment. However, the issues concerning the physical basis of temperature measurements, the formation of differential radiative heat transfer fluxes and the corresponding digital signals, the methods of temperature calculation with the uncertainty of the thermal emission coefficient, as well as the sources of uncertainty in temperature measurements are considered official information that is not widely disseminated. For users of thermographic equipment, such absence causes difficulties in the interpretation of measurement results in complex cases [3], e.g., with non-uniform heating, dynamic change of heat emission coefficient, presence of external illumination, etc. This is especially true for high-temperature thermography, where the techniques recommended by manufacturers of infrared thermal imaging equipment [1,4] to eliminate the influence of unknown factors are inapplicable due to the high temperature of objects. Therefore, in difficult conditions, it is necessary to apply thermographic techniques of different spectrum ranges [5,6,7,8,9], which is illustrated in Figure 1.

Thermal radiation of metals is generated by photons resulting from quantum processes in a thin surface layer of about 4 nm thick. Therefore, when the metal is heated to high temperatures, its surface roughness degree, its oxidation by atmospheric oxygen, presence of impurities, etc. can lead to a substantial change of spectral dependence of thermal radiation coefficient ε (λ) in comparison with the tabular values given in reference books. In addition, other factors related to the observation conditions of the heated surface and the parameters of the photodetector path of thermographic technology will also contribute to error in measuring temperature [8,9].

In large enterprises, when controlling processes with stably repeating parameters (for example, rolling mills), the deviation of the partial radiation temperature *T_r_* from the specified values is usually used, since the parameters of thermal processes are stable. In the case of new complex thermal processes, however, it is desirable to determine the real values of *T*. Therefore, when the ε*_eff_*, is uncertain, some authors suggest a more complex thermographic technique [10], using the measurement of thermal radiation in three [11], four [12], or even eight spectral bands [13].

Only low-cost thermographic techniques with stable methods of digital processing of registered thermal fields can have practical application in the control of high-temperature thermal processes. It can have two designs: (1) monoblock, when the thermograph chamber together with processing and indication unit are assembled in one inseparable case, and (2) combined, consisting of two units: the thermograph chamber and the processing, indication, recording, and storage device [14]. Communication between them is performed through a wired (USB, GIG-E) or wireless (Wi-Fi) interface. A serious advantage of the mobile two-unit design is the possibility of using modern small-size computing equipment as a processing device—laptops and tablets, which are convenient for recording, storing, and subsequent analysis of recorded thermograms and video recordings of complex dynamic processes. Thus, non-standard algorithms of processing the received thermal images optimized for a concrete task of temperature field control can be used.

In contrast to the thermographic technique of the LWIR range of the spectrum in thermography of the visible and NIR ranges, the spectral density of the brightness of the recorded thermal radiation *L* (*x*,*y*) is changed by several orders of magnitude [7,8,9,10,11]. Adjusting the illumination of the matrix of photodetectors by changing the f-number of the lens of the thermograph camera is unacceptable, since the solid angle of incidence of radiation on the light-sensitive elements changes, and there are deviations of the recorded signals from the calibration dependence. Therefore, it is necessary to introduce algorithms for the automatic selection of the exposure time of the frame τ*_ex_*, which can vary over a wide range, into the software of the photoreceiving path. At high temperatures and, accordingly, short exposure times, a noticeable effect of defects in the photodetector matrix used in the thermograph is manifested, which must be excluded. This requires the use of appropriate methods for carrying out calibration work, which makes it possible to take into account the peculiarities of the photodetector path and the noise properties of the used photodetector matrix.

When analyzing possible ways to solve the problems noted above, it is necessary to be guided by the basic principles of reducing the uncertainty of temperature measurement by thermographic technology in the high-temperature range. The following should be provided [15]:

- the ability to measure the true temperature *T* and estimate the effective emissivity ε*_eff_*;
- minimization of the uncertainty of the temperature measurement results achieved through the optimal choice of spectral sections for registration of thermal radiation and the elimination of the influence of the deviation of the calibration curves from the calculated dependences;
- determination of the maximum body temperature *T*_max_ and its dependence on time, as well as the possibility of video recording of the temperature field and its subsequent frame-by-frame viewing, which is necessary when monitoring complex heat engineering processes;
- invariance of the results of determining the maximum temperature *T*_max_ to changes in the size of the image of the monitored bodies;
- invariance of the measured values of *T*_max_ to nonstationarity of the noise dispersion of the used photodetector matrix, i.e., the dependence of its noise on the value of the incident thermal radiation flux.

To introduce these principles into the design and algorithms of software for high-temperature thermographic technology, it is necessary to carefully analyze the mechanisms of the influence of several factors on the uncertainty of the measured values of *T* [16] and the existing capabilities of photodetector matrices, optoelectronic technologies and algorithms for digital processing of recorded heat fluxes.

Therefore, the authors have tried to cover the principles of multispectral high-temperature thermographs functioning, using all available (or existing) arsenal of modern knowledge in character analysis and modelling of optoelectronic systems and digital technology.

## 2. Design and Quality Indicators of Thermographic Equipment

**Figure 2:**
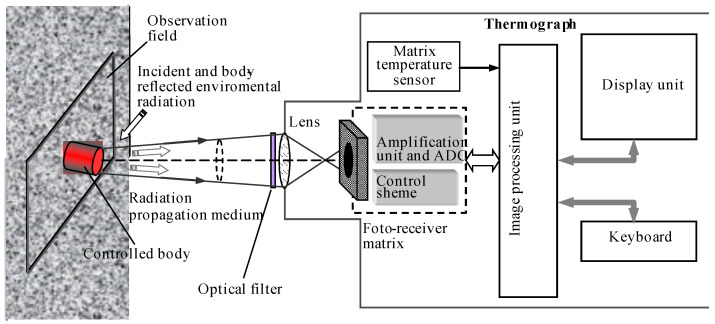
Scheme of temperature control with a thermograph based on a matrix of photodetectors and its functional diagram.

An idea of the composition of modern thermographs based on uncooled photodetector matrices is given by the diagram shown in Figure 2. The absence of moving mechanical elements, miniaturization of electronic components up to the inclusion of an ADC in the single-chip matrix of photodetectors, and mass production of compact lenses with high resolution makes it possible to create small-sized digital thermographs with the ability to quickly record the received thermograms and their primary analysis.

The currently available set of commercially available photodetector matrices of various spectral ranges makes it possible to create thermographic equipment with optimal parameters for measuring low, medium, and high temperatures. They usually include: a lens with a multilayer antireflection coating; an optical filter that forms the required spectral range and can be applied to the input window of the photodetector matrix; a built-in controllable shutter (in MWIR and LWIR ranges) to block the field of view in order to quickly correct the sensitivity of the thermograph elements, which allows ensuring uniformity of the thermograph sensitivity over the field of view; a matrix of photodetectors with a unit for reading signals, amplifying them and converting them into digital form; a display device or monitor; a control unit for the operation of the thermograph with the necessary controls; a block for processing the received images and displaying thermograms on the monitor screen; an interface for communication with external devices and a storage device for received data, which are usually stored in the form of files of a certain structure.

Since the assessment of the quality of thermographs based on the visually observed image is subjective, in the process of development of thermal imaging technology, a number of standard indicators (parameters and characteristics) have been developed, the measurement of which is carried out in the laboratory conditions [17,18,19]. They allow experts to predict how the camera will perform under real-world conditions. Standard indicators, a detailed description of which is given in [18,19], are usually divided into 8 groups. Of these, high-temperature thermography is usually used:

- accuracy parameters the root-mean-square value of the error with which the temperature can be measured;
- instantaneous field of view;
- frame formation time or frequency of their repetition;
- resolution.

## 3. Formation of a Digital Image of the Temperature Field

Thermographic equipment does not allow directly determining the distance to the object and therefore operates with signals that depend on two variables *x*, *y*, i.e., only with the dependence of the illumination *E* (*x*, *y*) of the object image and the background in the focal plane of the receiving lens. For incoherent radiation, the illumination *E* (*x*, *y*) in the image space is proportional to the brightness *L* (*x*_1_, *y*_1_) of conjugate points in the object space. When calculating the spectral illumination density *E* (λ) of the photosensitive element of the matrix with the area *s_ae_*, the spectral radiation flux density at the entrance pupil of the objective is usually determined

(1) $$\Phi_{ae}^{\mathrm{lens}}\left(\lambda \right)\approx L\left(\lambda \right)s_{ae}^{\mathrm{so}}\Omega =\frac{\pi D^{2}}{4f^{2}}{\left(1-\frac{f}{z}\right)}^{2}L\left(\lambda \right)s_{ae},$$

where $s_{ae}^{\mathrm{so}}$ is the area of the projection of this matrix element onto the surface of the observed object in the space of objects, Ω = π*D*^2^/(4*z*^2^) is the solid angle in which the radiation of the body from this area falls on the lens, *f* and *D* are the focal length and diameter of the entrance pupil of the lens, accordingly, and *z* is the distance from the surface of the observed object to the main optical plane of the lens. For Lambert emitters, their brightness *L* is proportional to their surface luminosity *M*, i.e., they are related by the expression *L* = *M*/π, which leads to a simple expression for the spectral illumination density of the photodetector matrix

(2) $$E\left(\lambda \right)=\frac{\Phi_{ae}^{lens}\left(\lambda \right)}{s_{ae}}=\frac{D^{2}}{4f^{2}}{\left(1-\frac{f}{z}\right)}^{2}M\left(\lambda \right)=KM\left(\lambda \right),$$

where the *K* coefficient takes into account the aperture of the lens and the distance *z* to the object

(3) $$K=\left(D^{2}/4f^{2}\right){\left(1-f/z\right)}^{2},$$

and is a dimensionless quantity, which can be called the coefficient of connection between the illumination of the thermograph matrix and the luminosity of the object at a unit transmittance of the lens τ*_l_* = 1. Then, the spectral flux entering the photosensitive element of the photodetector matrix through the lens is described by the expression

(4) $$\Phi_{ae}\left(\lambda \right)=\tau_{l}\frac{D^{2}}{4f^{2}}{\left(1-\frac{f}{z}\right)}^{2}M\left(\lambda \right)s_{ae}=\tau_{l}KM\left(\lambda \right)s_{ae}.$$

Averaged spectral slope of current conversion of photosensitive elements of photonic detector arrays

(5) $${\bar{S}}_{I}\left(\lambda \right)=\frac{\Delta I}{\Delta \Phi \left(\lambda \right)}=\frac{\eta_{ex}\left(\lambda \right)e\Delta \Phi \left(\lambda \right)}{hc/\lambda }\frac{1}{\Delta \Phi \left(\lambda \right)}=\frac{\eta_{ex}\left(\lambda \right)e\lambda }{hc},$$

takes into account the external quantum yield η*_ex_* (λ) of the photosensitive element, the power of the difference flux of radiation heat transfer ΔΦ (λ) carried by photons, and physical constants: the magnitude of the electron charge *e*, Planck’s constant *h*, and the speed of light in vacuum *c*. Multiplying ${\bar{S}}_{I}\left(\lambda \right)$ by Φ*_ae_* (λ), we obtain the current *I_ph_* generated by photons in the photosensitive elements of the matrix, which forms a charge *Q* = *I_ph_*⋅τ*_e_*. during the exposure of the frame τ*_e_*.

When calculating the signal *D_ij_* at the output of the ADC of the thermograph, coming from the *ij* elements of the photonic detector matrix, it is necessary to integrate the fluxes of monochromatic radiation falling on the photosensitive elements of the matrix within the used spectral region λ*_sw_*–λ*_lw_*

(6) $$D_{ij}\left(T,\tau_{э}\right)=\tau_{l}Ks_{ae}k_{ij}gk_{QD}\left[\frac{e}{hc}\int_{\lambda_{sw}}^{\lambda_{lw}}\tau_{of}\left(\lambda \right)\eta_{ex}\left(\lambda \right)M_{ij}\left(\lambda ,T\right)\lambda d\lambda \right]\tau_{e},$$

where τ*_of_* (λ) is the transmission coefficient of the optical filter, which, in the form of multilayer coatings, can be applied to the optical surfaces of the lens or the input window of the photodetector array, *k_QD_* is the conversion factor of the charge entering the input of the matrix reading unit into a digital code, and *g* is the gain of the amplifier built in before the ADC.

Modern arrays of photonic detectors have a sufficiently wide range of signal linearity and a system for subtracting the dark current; therefore, in further calculations, it is more convenient to use the rate of rise of digital counts obtained from (6) by dividing by the exposure time of the frame τ*_e_*,

(7) $$V_{ij}\left(T\right)=\tau_{l}Ks_{ae}k_{ij}gk_{QD}\left[\frac{e}{hc}\int_{\lambda_{sw}}^{\lambda_{lw}}\tau_{of}\left(\lambda \right)\eta_{ex}\left(\lambda \right)M_{ij}\left(\lambda ,T\right)\lambda d\lambda \right],$$

which is proportional to the magnitude of the photocurrents *I_ph_* generated by the photosensitive elements of the matrix, and, consequently, to the brightness of the observed objects. Since modern lenses have good spatial resolution, the effect of the spatial frequency transfer characteristic of the lens can be ignored.

**Figure 3:**
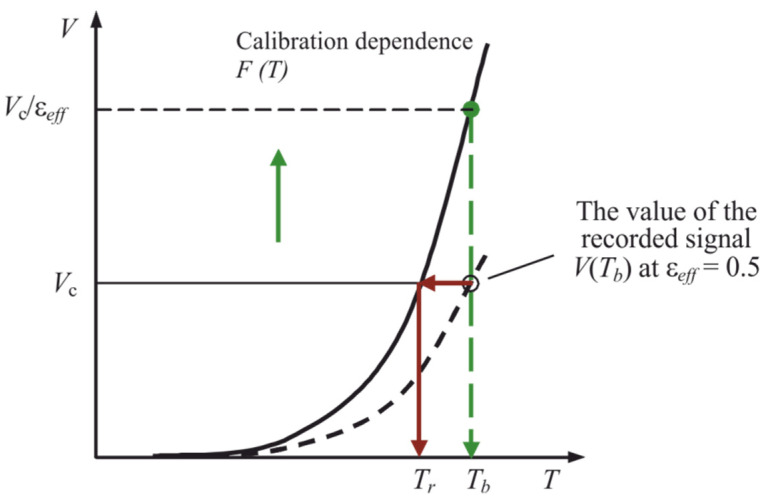
An approximate form of the calibration dependence *F* (*T*) (solid line) and the dependence of the recorded signal value on the body surface temperature *T_b_* at an effective thermal radiation coefficient ε*_eff_* = 0.5 (dashed line).

The program for processing the registered digital signals with the introduction of the effective value of the thermal radiation coefficient ε*_eff_* forms a palette image of the temperature field. The body temperature *T* is determined by comparing the rate of rise of digital signals *V_ji_* (7) divided by the effective thermal emissivity of the body ε*_eff_* with the calibration dependence *F* (*T*) (see Figure 3 [15]) obtained using a reference emitter—the black body model. When using the value ε*_eff_* = 1, the temperature of the partial radiation *T_r_* will be the result of measuring the temperature of real bodies.

To exclude the influence of the values of *g* and the distance *z* from the main plane of the lens of the thermograph to the body on the results of calculations of *T*, it is necessary to use the corrected measured values of the slew rates of digital signals

(8) $$V_{ij}^{cor}\left(T\right)=\frac{g_{c}K_{c}}{g_{m}K_{m}}V_{ij}\left(T\right),$$

where *g_c_*, *K_c_* and *g_m_*, *K_m_* are the values of the corresponding parameters when calibrating the thermograph and during temperature measurements, respectively.

**Figure 4:**
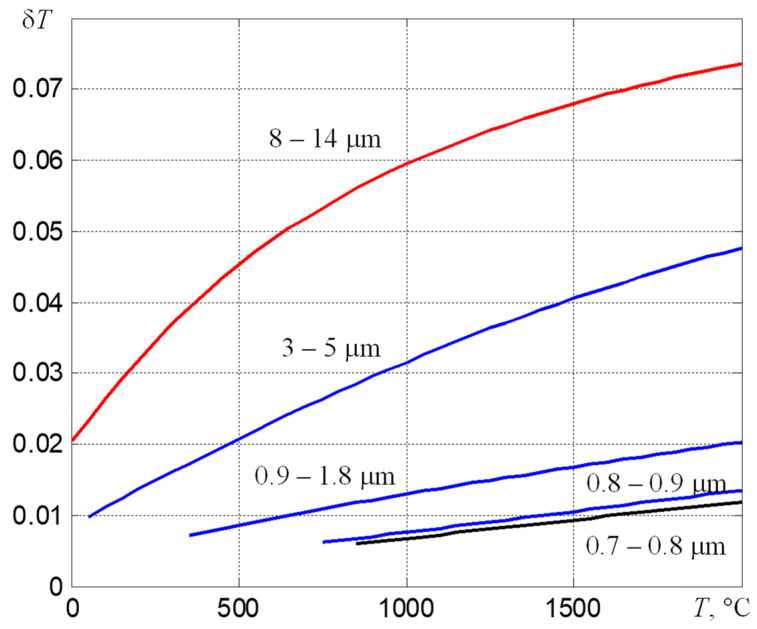
Temperature dependences of the relative errors δ*T* for determining the true temperature of bodies with thermal imagers of partial radiation, which use typical spectral ranges, at a relative uncertainty of the value of ε equal to 0.1.

To reduce the measurement error of *T*, it is necessary to use the optimal for a given range of measured temperatures *T*_min_–*T*_max_ spectral region of the thermal radiation registration. Its position on the wavelength axis λ is selected from the condition of the need to provide a sufficient signal-to-noise ratio at the maximum possible slope of the *V* (*T*) dependence, which leads to the dependences shown in Figure 4 [11]. It follows from them that, if it is necessary to measure temperatures *T* > 800 °C, the spectral range from 0.7 to 0.8 μm should be used.

## 4. Determining Temperature When Using Multiple Spectral Regions

When the thermograph uses one part of the spectrum, the automatic determination of the unknown effective coefficient of thermal radiation ε_eff_ is impossible, and to calculate the correct value of the temperature *T*, you need to know its value.

Registration of thermal radiation in two parts of the spectrum makes it possible to exclude the influence of only the emissivity of “gray” bodies on the results of determining the temperature, since for an unambiguous solution the system of two equations must contain only two unknown variables *T* and ε = const.

In the case of using three spectral regions, it becomes possible, when imposing an additional condition on the behavior of ε (λ), for example, a linear dependence ε (λ) = *a* + *b*λ with the obvious constraint 0 < ε(λ) < 1, to compose a system of three nonlinear equations and find *T* when solving it [20]. This approach is applicable in a narrow wavelength range, where the ε (λ) dependence is well described by a linear, exponential, hyperbolic, or other suitable dependence with two parameters.

A further increase in the number of spectral regions used requires registration of heat fluxes with high accuracy [21], which is achievable only in laboratory conditions.

The capabilities of modern multispectral photodetector arrays for measuring *T* from thermal radiation in several spectral regions are limited due to a noticeable overlap of spectral characteristics. For example, the Imec corporation advertises hyperspectral photodetector arrays that register radiation in 16 narrow areas in the 460–630 nm range [22]. However, their application in multispectral thermography is complicated by the presence of residual radiation transmission of the used optical filters on the wings of their spectral characteristics. Practical application in monitoring the temperature of bodies with an unknown spectral coefficient of thermal radiation ε (λ) can have simpler thermographs that record thermal radiation in three spectral regions.

### 4.1. Determination of T Using Three Narrow Spectral Regions

When analyzing the capabilities of high-temperature thermography with registration of thermal radiation in three narrow spectral regions with the wavelengths of centers λ_1_, λ_2_ and λ_3_, the Wien approximation can be used to simplify the calculated expressions. When imposing restrictions on the behavior of ε (λ), the exponential dependence is used ε (λ) = ε (λ_2_)∙exp[-*b*∙(λ − λ_2_)] = ε_2_∙exp(-*b*∙ΔΛ), where ε_2_ = ε (λ_2_) and ΔΛ = λ − λ_2_.

With a symmetric arrangement of λ_1_ and λ_3_ relative to λ_2_ and the presence of calibration dependences *F_i_* (*T*), the system of corresponding equations can be represented in the form

(9) $$\left\{ \begin{array}{l} f_{1}\left(T\right)=\varepsilon_{2}\frac{c_{1}}{\lambda_{1}^{5}}e^{-c_{2}/\left(\lambda_{1}T\right)-b\Delta \Lambda }=\varepsilon_{2}e^{-b\Delta \Lambda }F_{1}\left(T\right),\\ f_{2}\left(T\right)=\varepsilon_{2}\frac{c_{1}}{\lambda_{2}^{5}}e^{-c_{2}/\left(\lambda_{2}T\right)}\;\;\;=\varepsilon_{2}F_{2}\left(T\right),\\ f_{3}\left(T\right)=\varepsilon_{2}\frac{c_{1}}{\lambda_{3}^{5}}e^{-c_{2}/\left(\lambda_{3}T\right)+b\Delta \Lambda }=\varepsilon_{2}e^{b\Delta \Lambda }F_{3}\left(T\right), \end{array}\right.$$

Multiplying the first equation by the third and excluding *b*, and then ε_2_, one can obtain a nonlinear equation for finding *T*:

(10) $$\xi \left(T\right)=\frac{f_{2}^{2}}{F_{2}^{2}\left(T\right)}-\frac{f_{1}f_{3}}{F_{1}\left(T\right)F_{3}\left(T\right)}=0,$$

**Figure 5:**
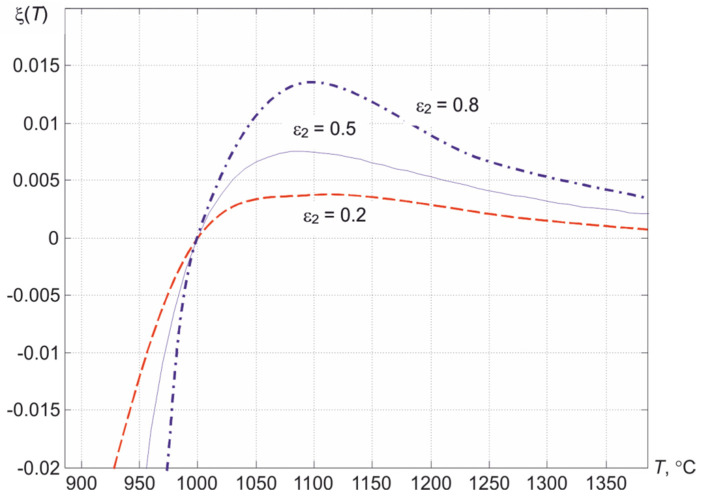
Dependence of the change in the value ξ on temperature at different ε_2_, λ_1_ = 0.6, λ_2_ = 0.75, λ_3_ = 0.9 μm and the value of the true temperature *T_real_* = 1000 °C.

Since the product *f*_1_*f*_3_ does not depend on *b*, the slope of the intersection of the abscissa of the curve ξ (*T*) for the exponential dependence ε (λ) depends only on the value of ε_2_, which is clearly seen from Figure 5 and confirms the analytical expression for the slope of the change in the function ξ (*T*) in point *T = T_real_*

(11) $${\frac{d\xi \left(T\right)}{dT}|}_{T=T_{real}}=\frac{c_{2}}{T_{{}_{real}}^{2}}\frac{2\Delta \Lambda^{2}}{\lambda_{1}\lambda_{2}\lambda_{3}}\varepsilon_{2}^{2}.$$

Using the expression for estimation the error in indirect measurements, one can find the mean-root-square deviation of the quantity ξ from the relative errors in measuring the fluxes δ*f*_1_, δ*f*_2,_ δ*f*_3_:

(12) $${\Delta \xi |}_{T=T_{и}}=\sqrt{{\left(\frac{\partial \xi }{\partial f_{1}}\right)}^{2}\Delta f_{1}^{2}+{\left(\frac{\partial \xi }{\partial f_{2}}\right)}^{2}\Delta f_{2}^{2}+{\left(\frac{\partial \xi }{\partial f_{3}}\right)}^{2}\Delta f_{3}^{2}}=\varepsilon_{2}^{2}\sqrt{\delta f_{1}^{2}+\delta f_{3}^{2}+4\delta f_{2}^{2}},$$

transforming which, it is possible to obtain the dependence of the relative error in determining the temperature δ*T* on δ*f*:

(13) $$\delta T=\frac{T_{real}^{}}{c_{2}}\frac{\lambda_{1}\lambda_{2}\lambda_{3}}{2\cdot \Delta \Lambda^{2}}\sqrt{6}\delta f_{2},$$

When using wavelengths λ_1_ = 0.6, λ_2_ = 0.75, and λ_3_ = 0.9 μm, we obtain the transfer coefficient of the relative error [21] when measuring a temperature of 1000 °C, equal to 1.95 or about 2. Then, at δ*f*_2_ = 0.002, we obtain δ*T* ≈ 0.004. When narrowing the used spectral region, i.e., at ΔΛ = 0.1 μm, we obtain the transfer coefficient of the random component of the error, equal to about 4.5, and δ*T* = 0.009 or about one percent.

Thus, the solution (10) of nonlinear Equation (9) with a sufficient distance ΔΛ between the used narrow spectral regions of registration of thermal radiation makes it possible to provide an acceptable error in determining the temperature *T*. In practice, materials for the formation of narrow spectral characteristics of optical filters with high attenuation on the wings, which could be applied on the surface of the photodetector matrix, are absent. The use of RGB matrices when measuring *T* is ineffective since ε (λ) of many materials in the visible region has dependences far from exponential. Therefore, one should consider the possibility of forming three parts of the spectrum, shifted in the NIR spectrum range when using commercially available RGB photodetector matrices.

### 4.2. Formation of Three Spectrum Regions, Shifted in the NIR Range

**Figure 6:**
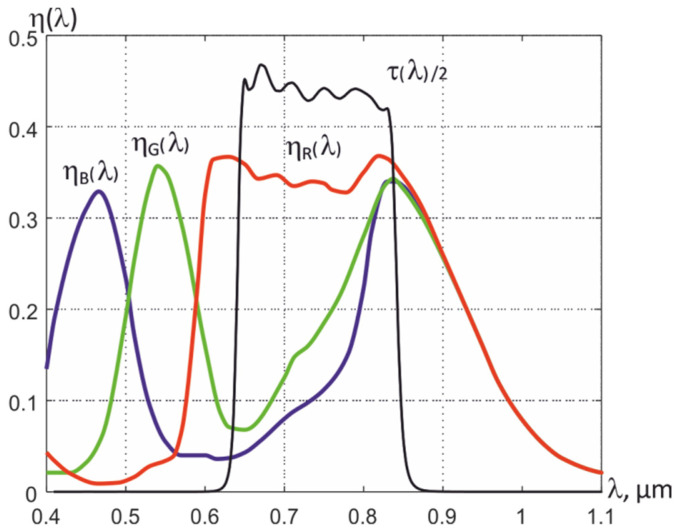
Spectral quantum efficiencies of RGB elements of the matrix of silicon photodetectors MT9V034C12STC and a 2-fold reduced transmission of the external band-pass optical filter τ (λ).

Analysis of the spectral quantum efficiency η*_ex_* (λ) of digital single-chip color CMOS matrices shows the possibility of forming three spectrum regions shifted in the NIR range when using an external optical filter. For example, the MT9V034C12STC matrix with a built-in 10-bit ADC has a good η*_ex_* (λ) value in the NIR spectrum range (see Figure 6), since it is also designed to work in low light at night. Since the freedom to choose the spectral regions for registering thermal radiation for RGB matrices is limited, in addition to an external optical filter, a combination of the received RGB signals must also be used.

**Figure 7:**
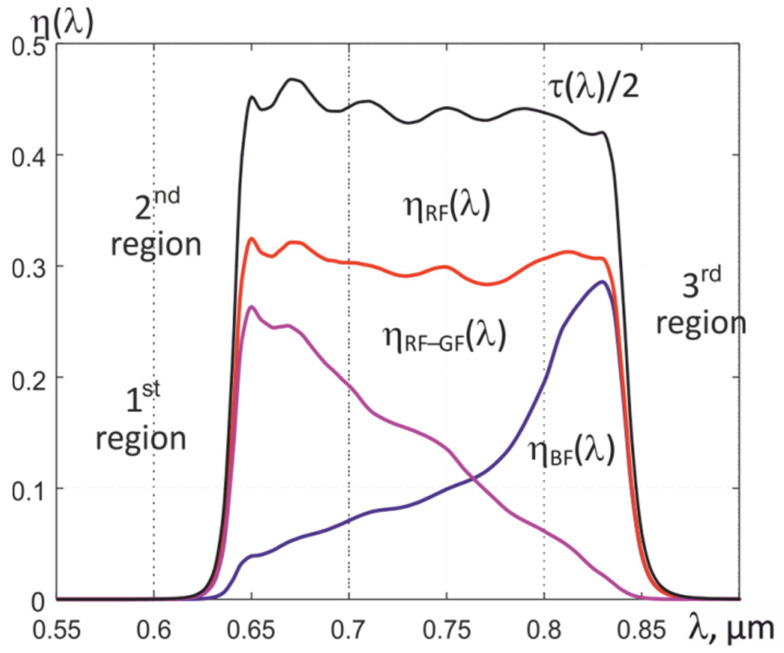
Resulting spectral quantum efficiencies in three spectral regions, formed using an external bandpass filter and a combination of recorded signals.

By using an external bandpass filter with the transmission of radiation in the NIR range, where an increase in the transmission of G and B filters of the Bayer mosaic is observed, and by calculating the difference between the signals formed by R and G by the light-sensitive elements of the matrix, it is possible to form the resulting quantum efficiencies η*_k_*(λ) in three spectral regions. For example, using a bandpass filter with a transmission in the 630–830 nm range and an MT9V034C12STC matrix, three dependences η (λ) can be formed, which are indicated in Figure 7 as 1st, 2nd, and 3rd sections. They completely overlap, but their effective wavelengths differ from each other, which makes it possible to use them to determine the temperature *T*, the effective value of the thermal emissivity ε*_eff_*_2_, and the conditional temperatures of partial radiation *T_rk_* in the formed spectral regions.

It should be understood that due to the small separation of the effective wavelengths of these sections, high accuracy of measurements of heat fluxes and the imposition of additional restrictions on the behavior of ε (λ) will be required in order to form a stable method for determining the temperature *T.* To ensure the required accuracy, it is necessary to take into account possible deviations of the calibration dependences from theoretically calculated. It will also be necessary to take into account the dependence of the statistical parameters of the noise of the photodetector channel of the three-spectral thermograph on the value of the incident fluxes of thermal radiation.

### 4.3. Temperature Determination Method

Proceeding similarly to the approach (9) already considered above, using narrow spectral regions, it is possible to compose a system of three nonlinear equations. The use of the exponential dependence ε (λ) = ε_2_∙exp [*b* (λ − λ_2_)] allows one to correctly describe the behavior ε (λ) of many bodies in a limited spectral region. For small values of the product *b*(λ − λ_2_) < 0.05, which is the case for many structural materials in practice, the exponential expression can be simplified by expanding the exponent in a power series and discarding terms with powers greater than unity, which gives ε (λ) = ε_2_ + *b* (λ − λ_2_). Then, assuming that *g* = 1 and *k_ij_* ≈ 1 and taking into account (7), we can compose the following system of equations

(14) $$\left\{ \begin{array}{l} V_{\mathrm{RF}-\mathrm{GF}}\left(T\right)=\tau_{l}Ks_{ae}k_{QD}\left\{\frac{e}{hc}\int_{\lambda_{sw}}^{\lambda_{lw}}\tau_{of}\left(\lambda \right)\eta_{\mathrm{RF}-\mathrm{GF}}\left(\lambda \right)\left[\varepsilon_{2}+b\left(\lambda -\lambda_{2}\right)\right]M\left(\lambda ,T\right)\lambda d\lambda \right\},\\ V_{\mathrm{RF}}\left(T\right)=\tau_{l}Ks_{ae}k_{QD}\left\{\frac{e}{hc}\int_{\lambda_{sw}}^{\lambda_{lw}}\tau_{of}\left(\lambda \right)\eta_{\mathrm{RF}}\left(\lambda \right)\left[\varepsilon_{2}+b\left(\lambda -\lambda_{2}\right)\right]M\left(\lambda ,T\right)\lambda d\lambda \right\},\\ V_{\mathrm{BF}}\left(T\right)=\tau_{l}Ks_{ae}k_{QD}\left\{\frac{e}{hc}\int_{\lambda_{sw}}^{\lambda_{lw}}\tau_{of}\left(\lambda \right)\eta_{\mathrm{BF}}\left(\lambda \right)\left[\varepsilon_{2}+b\left(\lambda -\lambda_{2}\right)\right]M\left(\lambda ,T\right)\lambda d\lambda \right\}, \end{array}\right.$$

where *M* (λ, *T*) is the spectral density of the surface luminosity of an absolutely black body described by the corresponding Planck’s formula.

After integration, (14) can be represented in a more convenient form for further calculations

(15) $$\left\{ \begin{array}{l} V_{\mathrm{RF}-\mathrm{GF}}\left(T\right)=\varepsilon_{2}F_{\mathrm{RF}-\mathrm{GF}}\left(T\right)+bq_{\mathrm{RF}-\mathrm{GF}}\left(T\right)=\left[\varepsilon_{2}+b\mu_{\mathrm{RF}-\mathrm{GF}}\left(T\right)\right]F_{\mathrm{RF}-\mathrm{GF}}\left(T\right),\\ V_{\mathrm{RF}}\left(T\right)=\varepsilon_{2}F_{\mathrm{RF}}\left(T\right)+bq_{\mathrm{RF}}\left(T\right)=\left[\varepsilon_{2}+b\mu_{\mathrm{RF}}\left(T\right)\right]F_{\mathrm{RF}}\left(T\right),\\ V_{\mathrm{BF}}\left(T\right)=\varepsilon_{2}F_{\mathrm{BF}}\left(T\right)+bq_{\mathrm{BF}}\left(T\right)=\left[\varepsilon_{2}+b\mu_{\mathrm{BF}}\left(T\right)\right]F_{\mathrm{BF}}\left(T\right), \end{array}\right.$$

where *F*_RF-GF_ (*T*), *F*_RF_ (*T*), and *F*_BF_(*T*) correspond to the calibration dependences of the thermograph, which are determined experimentally using the black body model, *q*_RF-GF_ (*T*), *q*_RF_(*T*), and *q*_BF_ (*T*) are the integrals obtained by calculation and determining the dependences of these integrals on the temperature at a unit value of the coefficient *b* of the used dependence ε (λ) in the corresponding spectral region, and the dependences μ_RF-GF_ (*T*), μ_RF_ (*T*), and μ_BF_ (*T*) are integrals *q*_RF-GF_ (*T*), *q*_RF_ (*T*), and *q*_BF_ (*T*), normalized to the corresponding calibration dependences.

**Figure 8:**
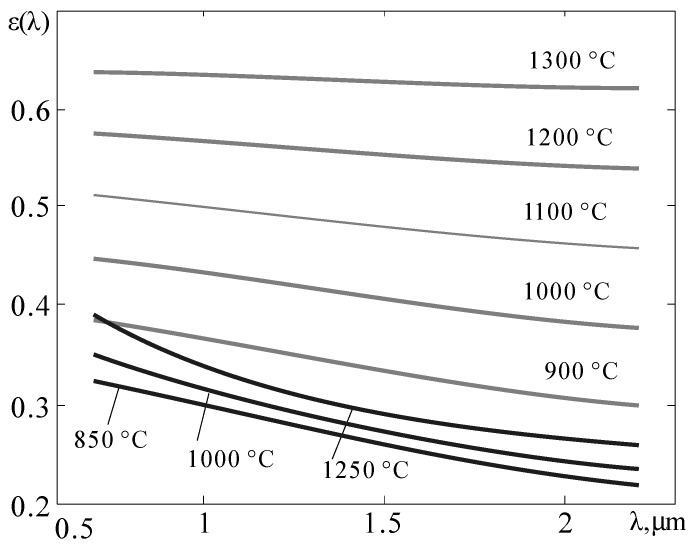
Changes in the ε (λ) dependence of pure iron when it is heated in vacuum [18] (black lines) and during gradual oxidation with atmospheric oxygen (gray lines).

Due to the overlap of the used spectral regions and the inevitable spread of the recorded values of *V*_RF_-_GF_, *V*_RF_, and *V*_BF_ due to the presence of noise, the numerical solution of system (15) gives an unacceptable spread of the obtained values of *T*. To obtain a more stable solution, an additional constraint on the behavior ε (λ) in the form of a functional connection *b* = γ∙*p* (ε_2_) between the slope coefficient *b* of the spectral dependence ε (λ) of the controlled surface, the value of the coefficient γ and the value of ε_2_ in the form of a polynomial of the second degree with given values of the coefficients *p*_2_, *p*_1_, and *p*_0_. The feasibility of introducing this functional bond is due to an increase in the values of ε (λ) when the controlled surface is heated, which occurs due to its oxidation with atmospheric oxygen. As an example, Figure 8 shows the dependences ε (λ) of clean (black lines) and oxidized (gray lines) steel surface. Oxidation of the surface leads to a decrease in the slope coefficient b from the maximum possible value to close to zero.

**Figure 9:**
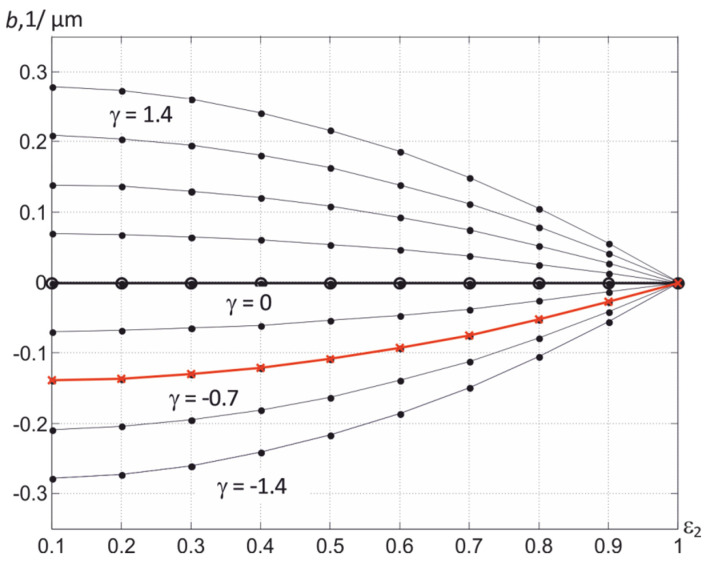
Family of dependences *b* (γ, ε_2_) when using expression (16) and changing γ in the range from minus 1.4 to 1.4 μm^−1^ with a step of 0.35 μm^−1^.

Several sets of coefficients *p*_2_, *p*_1_, and *p*_0_ of the used polynomial can be proposed. For example, when describing the relationship between the values of *b* and ε_2_ for bodies with possible ranges of variation of the thermal radiation coefficient 0.05 < ε_2_ < 1, one can use a power polynomial in the form

(16) $$b=\gamma \left(-\frac{80}{361}\varepsilon_{2}^{2}+\frac{8}{361}\varepsilon_{2}+\frac{72}{361}\right),$$

which describes a smooth decrease in the absolute value of ∣*b*∣ with an increase in ε_2_ in the process of surface oxidation with atmospheric oxygen. The corresponding graphical dependencies are shown in Figure 9. The curves in the lower half-plane, where γ < 0, refer to metals, and in the upper, to dielectrics. Since the spectral dependences of the thermal radiation coefficients of most structural materials are known [23], the value of γ is calculated in advance from the value of *b* and ε_2_ of pure unoxidized material, which provides an adequate calculation of *b* when the value of ε_2_ changes during oxidation with atmospheric oxygen. For tungsten, the value of γ is γ = −1.07, and for ferrous metals, γ ≈ −0.5. Comparing Figure 8 and Figure 9, it can be seen that the slope coefficient *b* during steel oxidation tends to zero as ε_2_ grows.

The use of dependence (16), i.e., the involvement of a priori information on the possible behavior ε (λ) of the surface of the controlled body, allows one to significantly reduce the requirements for the accuracy of measuring heat fluxes and to use overlapping spectral regions in determining the temperature *T*. It should be noted that an increase in the distance between the used spectrum regions, on the one hand, reduces the requirements for the magnitude of measurement errors, but, on the other hand, due to the deviation of the behavior ε (λ) of bodies in the visible region from the linear law leads to violation of the condition ε (λ) = ε_2_ + *b* (λ − λ_2_), which, in turn, leads to an increase in the error in determining *T*. This is the main reason that prevents the effective use of the R, G, and B regions of the visible spectrum in high-temperature thermography.

When using (16) and smoothing *V*_RF-GF_ (*T*), *V*_RF_, and *V*_BF_ using a median digital filter that suppresses impulse emissions of defective elements of the used photodetector matrix, the system of Equation (15) takes the form

(17) $$\left\{ \begin{array}{l} {\bar{V}}_{\mathrm{RF}-\mathrm{GF}}\left(T\right)/F_{\mathrm{RF}-\mathrm{GF}}\left(T\right)=\varepsilon_{2}+\gamma \left(-\frac{80}{361}\varepsilon_{2}^{2}+\frac{8}{361}\varepsilon_{2}+\frac{72}{361}\right)\mu_{\mathrm{RF}-\mathrm{GF}}\left(T\right),\\ {\bar{V}}_{\mathrm{RF}}\left(T\right)/F_{\mathrm{RF}}\left(T\right)=\varepsilon_{2}+\gamma \left(-\frac{80}{361}\varepsilon_{2}^{2}+\frac{8}{361}\varepsilon_{2}+\frac{72}{361}\right)\mu_{\mathrm{RF}}\left(T\right),\\ {\bar{V}}_{\mathrm{BF}}\left(T\right)/F_{\mathrm{BF}}\left(T\right)=\varepsilon_{2}+\gamma \left(-\frac{80}{361}\varepsilon_{2}^{2}+\frac{8}{361}\varepsilon_{2}+\frac{72}{361}\right)\mu_{\mathrm{BF}}\left(T\right). \end{array}\right.$$

Subtracting the first equation from the third equation of system (16), we can obtain a quadratic equation for finding the dependence of ε_2_ on the estimate of the temperature T *:

(18) $$\varepsilon_{{}_{2}}^{2}\left(T^{*}\right)-0,1\varepsilon_{2}\left(T^{*}\right)-0,9+\frac{361}{80}\frac{{\bar{V}}_{\mathrm{BF}}/F_{\mathrm{BF}}\left(T^{*}\right)-{\bar{V}}_{\mathrm{RF}-\mathrm{GF}}/F_{\mathrm{RF}-\mathrm{GF}}\left(T^{*}\right)}{\gamma \left[\mu_{\mathrm{BF}}\left(T^{*}\right)-\mu_{\mathrm{RF}-\mathrm{GF}}\left(T^{*}\right)\right]}=0.$$

Choosing a positive solution to this equation,

(19) $$\varepsilon_{2}=0.05+\sqrt{0.9025-\frac{361}{80}\frac{{\bar{V}}_{\mathrm{BF}}/F_{\mathrm{BF}}\left(T^{*}\right)-{\bar{V}}_{\mathrm{RF}-\mathrm{GF}}/F_{\mathrm{RF}-\mathrm{GF}}\left(T^{*}\right)}{\gamma \left[\mu_{\mathrm{BF}}\left(T^{*}\right)-\mu_{\mathrm{RF}-\mathrm{GF}}\left(T^{*}\right)\right]}}.$$

and substituting it into the second equation of system (17), we obtain a nonlinear equation for finding the desired temperature value

(20) $$\xi \left(T^{*}\right)=\varepsilon_{2}\left(T^{*}\right)+\gamma \left[-\frac{80}{361}\varepsilon_{2}^{2}\left(T^{*}\right)+\frac{8}{361}\varepsilon_{2}\left(T^{*}\right)+\frac{72}{361}\right]\mu_{\mathrm{RF}}\left(T^{*}\right)-\frac{{\bar{V}}_{\mathrm{RF}}}{F_{\mathrm{RF}}\left(T^{*}\right)}=0.$$

The disadvantage of the obtained solution is the uncertainty of the calculated values of ε_2_ at values of γ close to zero. To eliminate it for small values of γ, one can use the smallness of the product *b*μ_RF_ (T*) in comparison with the value of ε_2_ (T*). This makes it possible to approximate the dependence ε_2_ (T*) as a ratio ${\bar{V}}_{\mathrm{RF}}/F_{\mathrm{RF}}\left(T^{*}\right)$ or, more precisely, ${\bar{V}}_{\mathrm{RF}}/F_{\mathrm{RF}}\left(T^{*}\right)-b\mu_{2}\left(T^{*}\right)$, which makes it possible to obtain an approximate expression for the dependence *b* (T^*^)

(21) $$b\left(T^{*}\right)\approx \gamma \left\{-\frac{80}{361}\frac{{\bar{V}}_{\mathrm{RF}}^{2}}{F_{\mathrm{RF}}^{2}\left(T^{*}\right)}+\frac{8}{361}\frac{{\bar{V}}_{\mathrm{RF}}}{F_{\mathrm{RF}}\left(T^{*}\right)}+\frac{72}{361}\right\}.$$

Substituting (21) into the first and second equations of system (17) and forming their difference, we obtain the resulting equation for determining *T*

(22) $${\bar{V}}_{\mathrm{RF}-\mathrm{GF}}/F_{\mathrm{RF}-\mathrm{GF}}\left(T^{*}\right)-{\bar{V}}_{\mathrm{BF}}/F_{\mathrm{BF}}\left(T^{*}\right)+b\left(T^{*}\right)\left[\mu_{\mathrm{BF}}\left(T^{*}\right)-\mu_{\mathrm{RF}-\mathrm{GF}}\left(T^{*}\right)\right]=0.$$

To linearize Equation (15), we can multiply it by the ratio ${\bar{V}}_{\mathrm{RF}}/F_{\mathrm{RF}}\left(T^{*}\right)$, which greatly simplifies the search for a numerical solution *T*:

(23) $$\xi \left(T^{*}\right)=\frac{{\bar{V}}_{\mathrm{RF}}}{F_{\mathrm{RF}}\left(T^{*}\right)}\left\{\frac{{\bar{V}}_{\mathrm{RF}-\mathrm{GF}}}{F_{\mathrm{RF}-\mathrm{GF}}\left(T^{*}\right)}-\frac{{\bar{V}}_{\mathrm{BF}}}{F_{\mathrm{BF}}\left(T^{*}\right)}+b\left(T^{*}\right)\left[\mu_{\mathrm{BF}}\left(T^{*}\right)-\mu_{\mathrm{RF}-\mathrm{GF}}\left(T^{*}\right)\right]\right\}=0.$$

**Figure 10:**
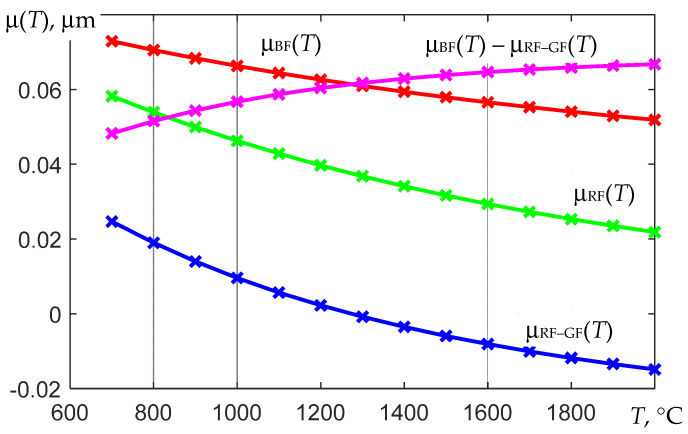
Calculated temperature dependences of the coefficients μ_RF-GF_ (*T*), μ_RF_ (*T*), μ_BF_ (*T*), and μ_BF_ (*T*) − μ_RF-GF_ (*T*) for the spectral quantum efficiencies shown in Figure 7.

**Figure 11:**
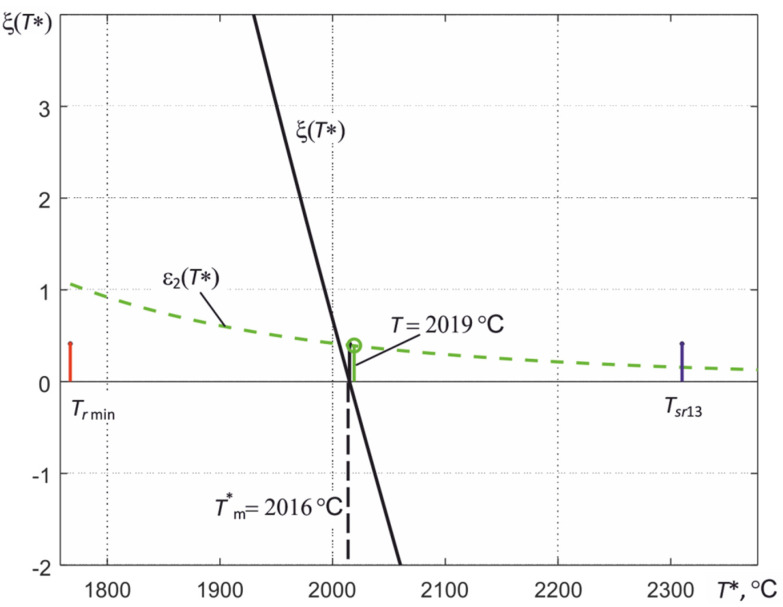
Illustration of the process of finding a solution to Equation (22) when determining the temperature of the region of maximum heating of the tungsten tape of the standard incandescent lamp SI10-300.

Examples of the calculated dependences μ_RF-GF_ (*T*), μ_RF_ (*T*), and μ_BF_ (*T*) for the spectral regions are shown in Figure 7, and illustrations of the numerical solution of the nonlinear equation using (23) are shown in Figure 10 and Figure 11.

In the numerical solution of Equation (19) or (22), the range in which the solution is sought is set, i.e., the estimate of the temperature T*. As can be clearly seen from the illustration of the process of finding a solution shown in Figure 9, the low-temperature limit of this range is determined by the minimum of the three temperatures of partial radiation *T_r_*_min_, recorded by the thermograph. The high-temperature limit for metals (γ < 0) is determined by the temperature of the spectral ratio for the first and third spectral regions *T_sr_*_13_, which is calculated by the thermograph when solving the equations. For dielectrics (γ > 0), the *T_sr_*_13_ value is usually less than the true surface temperature, so for them, the high-temperature boundary is chosen equal to 1.15∙*T_sr_*_13_.

Figure 11 shows an illustration of the process of finding a solution to Equation (22) using signals recorded by a three-spectral thermograph based on an MT9V034C12STC array of photodetectors. The spectral regions in which the thermograph records the thermal radiation correspond to those shown in Figure 7. The thermograph was calibrated according to the emission of the Mikron M390 black body model. The relative error in measuring *T* was 0.5% of the measured value *T*. This makes it possible to measure the temperature of bodies with a known value of ε*_eff_* with a relative error of about 0.5%. When measuring the temperature of the tungsten tape of the standard incandescent lamp SI10-300 in advance for the second spectral region used by the thermograph, the dependence of the effective thermal radiation coefficient of tungsten ε*_eff_*_2_ (*T*) was calculated taking into account the transmission of the quartz window of this lamp. By introducing the obtained value of ε*_eff_*_2_ (*T_m_*) into the thermograph and adjusting the current through the tungsten tape of the lamp, we achieved that the temperature of the tungsten tape in the region of its maximum heating corresponded to *T_m_*. For the case shown in Figure 11, the *T_m_* value measured in the second spectral region was 2019 °C.

The measured signals *V*_RF_-_GF_, *V*_RF_, and *V*_BF_, due to the presence of noise and the deviation of the thermograph characteristics from the calculated values, have fluctuations, which lead to a scatter of the obtained values of the measured temperature T^*^_m_ when solving Equation (22). For the example shown in Figure 11, the obtained estimate of T^*^_m_ when using the value γ = −1.07 µm^−1^ turned out to be approximately 3 °C lower than *T_m_*. The root-mean-square deviation of the temperature estimation of the most heated region of the tungsten tape during the averaging was about 1.5% of the measured value *T*. The dashed line in Figure 11 shows how the value of ε_2_(T*) changes in the process of finding a solution.

**Figure 12:**
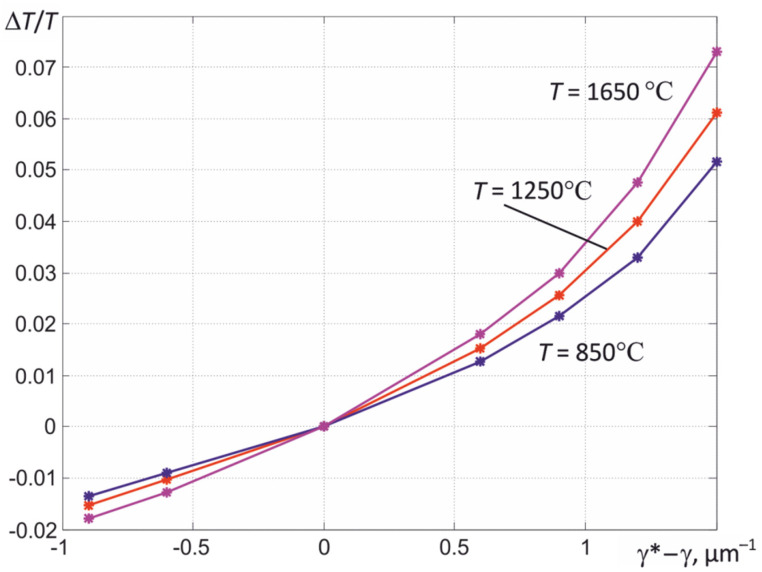
The results of evaluating the relative error in determining the temperature when the used value γ* deviates from its true value γ.

The error in determining the temperature in the described way is also affected by the error in setting the value of the coefficient γ. The performed modeling shows that, for metals, the relative errors in determining the true temperature Δ*T*/*T* with deviations of the used value γ* from its true value γ by ±40% in practice, as shown in Figure 12, do not exceed 0.015. This is due to the weak influence of the slope coefficient *b* of the spectral dependence ε (λ) on the obtained values of ε*_eff_*. Such errors are permissible when solving the overwhelming majority of practical problems of contactless temperature measurement of various heat engineering processes.

The described method for determining the temperature with an unknown coefficient of thermal emissivity makes it possible to determine ε*_eff_*_2_ = *M*(*T_r_*_2_)/*M*(T^*^_m_) from the obtained values of *T_r_*_2_ and T^*^_m_. Using the obtained value of ε*_eff_*_2_, one can switch to the usual mode of temperature measurement taking into account ε*_eff_*_2_, which reduces the variation in readings. As follows from the dependencies shown in Figure 4, the relative error in determining the temperature in the range from 0.7 to 0.8 μm will be an order of magnitude smaller than the relative error in determining ε*_eff_*_2_. This indicates the possibility of using three-spectral thermographs when setting up and periodically monitoring complex high-temperature technological processes. However, in order to reduce the error in determining ε*_eff_*_2_, it is necessary to use algorithms for averaging the recorded fields of thermal radiation, both in time and over the region of maximum heating.

## 5. Tehnique of Averaging Signals over the Area of Maximum Heating

**Figure 13:**
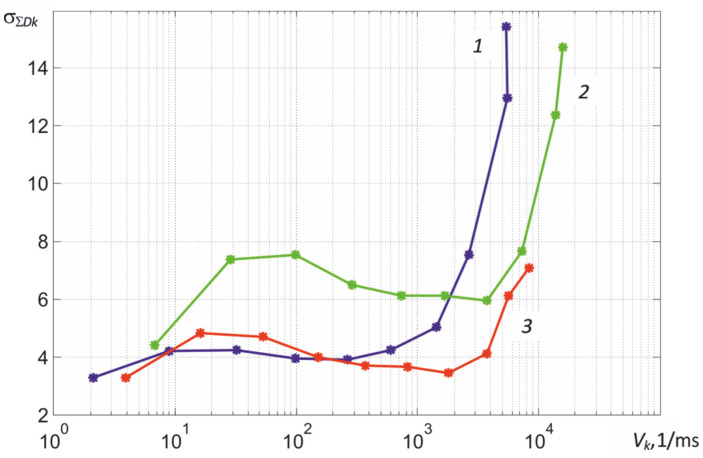
Dependences of the RMS of the total noise of the elements of the MT9V034C12STC matrix of the thermograph camera in ADC readings on the value of the recorded signals *V_k_* in the first, second, and third spectral regions at the focal length of the lens *f_l_* = 50 mm and the ratio *D*/*f_l_* = 1/16.

An unpleasant feature of MT9V034C12STC CMOS sensors with a global exposure mode is a rapid increase in the root mean square deviation of the total noise at high thermal radiation fluxes and, accordingly, small frame exposure times τ*_e_*, which is illustrated in Figure 13. At high fluxes, dots begin to appear on the resulting image, the brightness of which is noticeably different from the brightness of neighboring pixels. Moreover, their number increases with decreasing τ*_e_*. Matrix elements corresponding to these points are usually called “faulty”, since an increase in the currents generated by them is caused by the presence of defects in the structures of the insulator and the corresponding keys of the multiplexer. Therefore, manufacturers of high-temperature thermographs based on CMOS structures divide the range of measured temperatures into a number of subranges that differ in different values of the lens aperture *D*/*f_l_*. Partially fluctuating emissions of defective elements are excluded by median filtration. However, if it is necessary to control the heat engineering processes simultaneously in one wide temperature range, it is necessary to take into account the influence of the nonstationarity of the matrix noise and carefully take into account the existing deviations of the real characteristics of the thermograph from the calculated ones.

### 5.1. Ensuring the Invariance of the Measured Values of the Maximum Temperature T_max_ to the Image Size and the Nonstationarity of the Matrix Noise

In high-temperature thermography, the sizes of objects whose temperature must be controlled can be varied, and the temperature distribution over their surface is almost always inhomogeneous. In the process of heating objects, their temperature field can change quite quickly. Therefore, it is practically impossible to conduct a visual assessment of the temperature values from the image of the obtained temperature field. We have to implement the function of determining the maximum body temperature *T*_max_ into the software of high-temperature thermographs. The algorithms used in it must ensure that the above principles are followed to increase the reliability of temperature measurements. In particular, they must ensure the invariance of the results of determining the maximum temperature *T*_max_ to a change in the size of the image of the monitored bodies and the nonstationarity of the noise dispersion of the used photodetector matrix, i.e., the dependence of its noise on the value of the incident thermal radiation flux.

Neglect of these principles, when observing a sequence of frames, leads to a scatter of the obtained *T*_max_ readings and an abrupt displacement of the point with the maximum temperature value over the zone of greatest heating of the generated temperature field. The reason for this phenomenon is due to the statistics of the number of registered photons, which obeys the Poisson distribution, the intrinsic noise of the thermograph chamber, and fluctuations in the temperature of the surface of the heated body. Fluctuations in surface temperature are caused by its interaction with colder convection air currents, which are formed due to the temperature difference between the heated body and the air temperature.

**Figure 14:**
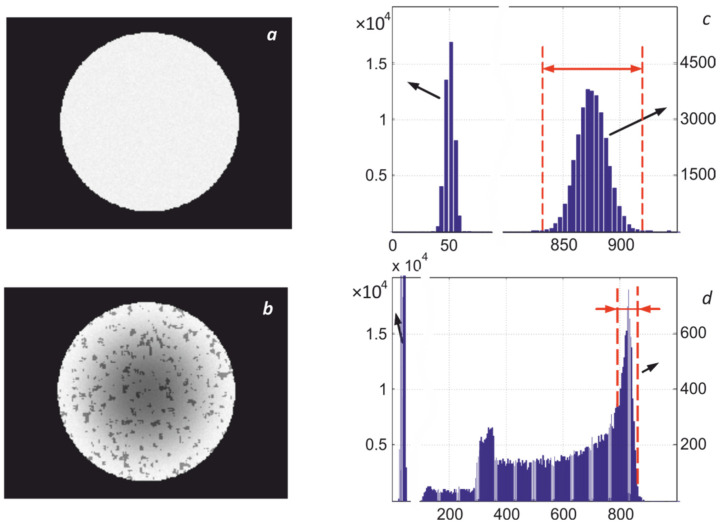
Examples of thermal images with uniform (**a**) and non-uniform (**b**) temperature distribution over the surface and their corresponding histograms (**c**,**d**).

**Figure 15:**
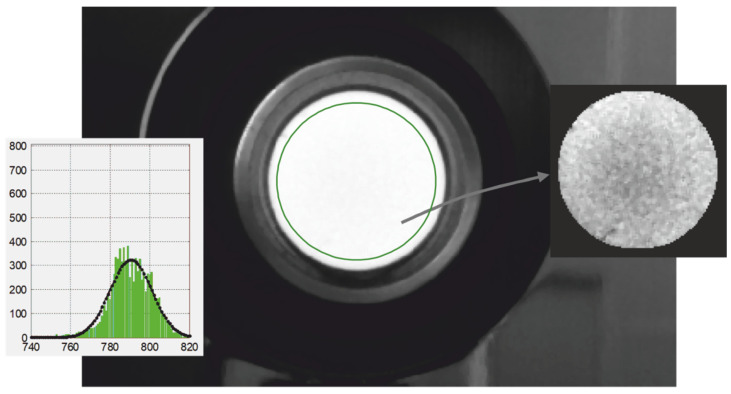
The image of the output diaphragm of the M390 black body model recorded by the thermograph in the second spectral region at a temperature of its radiating core of 800 °C and an exposure time of 130 ms, as well as an image of its central zone with a range of *D*_2_ signals from 760 to 820 ADC counts and the corresponding histogram approximated using normal distribution.

The presence of inhomogeneity of heating leads to distortion of the high-temperature mode of the histogram of the thermal image, which is illustrated in Figure 14. Even the radiating cavity of black body models can have a temperature gradient of several degrees, which distorts the corresponding histogram, as shown in Figure 15. The discreteness of the obtained digital images and the presence of fluctuations require averaging the obtained readings, which are used to calculate the maximum temperature. This raises the problem of optimal selection of image elements or pixels involved in averaging.

As seen in Figure 14d, when determining the maximum temperature *T*_max_, it is necessary to calculate the averaged values of digital readings ${\bar{D}}_{k}^{*}\left(T_{\max}\right)$ on the high-temperature slopes of the right wing of the thermal image histograms in the used spectral regions. The determination technique of ${\bar{D}}_{k}^{*}\left(T_{\max}\right)$ can be based on the existing relationship between the average values of the recorded signals and their variance

(24) $$\sigma_{\Sigma Dk}^{2}\approx gk_{QD}^{}e{\bar{D}}_{k}+{\bar{D}}_{k}^{2}\sigma_{n}^{2}+g^{2}\sigma_{Dread}^{2},$$

where the first term of the sum describes the variance of the radiation and shot noises, the second describes the geometric noises (caused by the inhomogeneity of the sensitivity of the matrix elements) with $\sigma_{n}^{2}$ variance, and the third describes the noises of the readout unit of the matrix with variance $\sigma_{Dread}^{2}$. Expression (24) determines the algorithm for choosing the required averaging interval.

### 5.2. Method for Obtaining Averaged Values ${\bar{V}}_{k}^{*}$ on the High-Temperature Slope of Histograms

Since the Poisson distribution used to describe radiation and shot noises is close to the normal distribution with a sufficiently large number of registered photons, the averaging interval can be chosen to be equal to $6\sigma_{\Sigma Dk}^{}\left({\bar{V}}_{k}^{*}\right)$ in the determination of ${\bar{V}}_{k}^{*}$. Additionally, the algorithm should include a method for clearing the histograms $y_{hk}\left(n\right)=H_{k}\left[x_{hk}\left(n\right)\right]$ from single outliers on its right wing, taking into account the dependence $\sigma_{\Sigma Dk}^{}\left({\bar{V}}_{k}\right)$.

**Figure 16:**
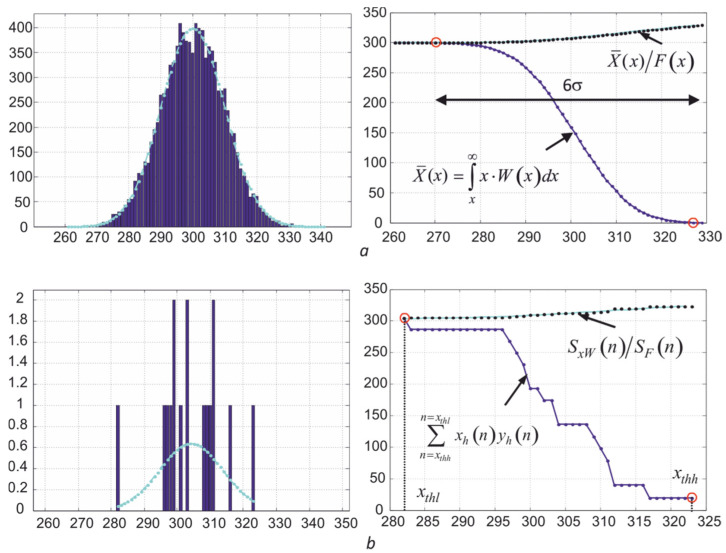
Histograms *H* (*D*) and the dependence of the estimation results ${\bar{D}}_{}^{*}$ on the values of the bin *H* (*D*) at *k_l_* = *k_h_* = 3 for different numbers of image pixels involved in averaging (*N* = 10^4^ (**a**) and *N* = 16 (**b**)) at $\bar{D}=300$ and σ = 10, where $k_{l}=\left|\left(D_{thl}-\bar{D}\right)/\sigma \right|$, and $k_{h}=\left(D_{thh}-\bar{D}\right)/\sigma$.

Taking into account the described factors, the method of determination ${\bar{V}}_{k}^{*}$ consists of the following sequence of actions:

(1) After receiving the frame, histograms are calculated for all three of its layers $y_{hk}\left(n\right)=H_{k}\left[x_{hk}\left(n\right)\right]$ with the width of pockets or bins equal to one or one level of the ADC conversion range.
(2) Carry out operations to clean the high-temperature sections of the histograms from single noise emissions, assessing the probability of their occurrence, taking into account the current values of the standard deviation equal to $\sigma_{\Sigma Dk}^{}\left[V_{k\max}-3\sigma_{\Sigma Dk}^{}\left(V_{k\max}\right)\right]$. This ensures the correct position on the histogram of the averaging intervals with width $6\sigma_{\Sigma Dk}^{}\left(V_{k\max}\right)$, where *V_k_*_max_ are the rightmost points of the histograms after clearing.
(3) Determine the left boundaries of the areas of integration of the histograms $x_{thlk}=x_{hk\max}-6\sigma_{\Sigma Dk}^{}\left(V_{k\max}\right)$.
(4) Calculate the dependences of two sums: the dependence of the area of the histogram section $S_{Fk}\left(n\right)=\sum_{n=x_{thh}}^{n=x_{thl}}y_{h}\left(n\right)$ and the sum $S_{xWk}\left(n\right)=\sum_{n=x_{thh}}^{n=x_{th}{}_{l}}x_{h}\left(n\right)y_{h}\left(n\right)$ on the current number *n* of the histogram bins. Moreover, they are summed from right to left, which starts from the maximum values of *n*. These dependences correspond to the integrals $F(x)=\int_{x}^{\infty }W\left(x\right)dx$ and $\bar{X}(x)=\int_{x}^{\infty }x\cdot W\left(x\right)dx$, where *W*(*x*) is the normal distribution.
(5) Normalize dependencies $S_{xWk}\left(n\right)$ by dividing them by $S_{Fk}\left(n\right)$, which corresponds to the relationship $\bar{X}(x)/F\left(x\right)=\int_{x}^{\infty }x\cdot W\left(x\right)dx/\int_{x}^{\infty }W\left(x\right)dx$. As seen in Figure 16, the values of the elements of the series $S_{xW}\left(n\right)/S_{F}\left(n\right)$ gradually decrease to the desired average value $\bar{D}$ when the point *x* moves from the maximum value *D* to the left, since the value of the integral is in the denominator of the ratio $\int_{\bar{D}-3\sigma_{\Sigma D}}^{\infty }W\left(x\right)dx\approx 1$. When moving to the left, the ratio $S_{xW}\left(n\right)/S_{F}\left(n\right)$ has a small steepness, determined by the value of σ, which makes it possible to obtain a slightly biased estimate $\bar{D}$ with a small number of pixels involved in averaging.
(6) Find an estimate of the mean values ${\bar{V}}_{k}^{*}={\bar{D}}_{k}^{*}/\tau_{e}$, where ${\bar{D}}_{k}^{*}$ is determined by enumerating the values of the dependencies $S_{xWk}\left(n\right)/S_{Fk}\left(n\right)$ and $S_{xWk}\left(n\right)$ starting from the maximum value of *n* to their intersection. The value $S_{xWk}\left(n_{l}\right)/S_{Fk}\left(n_{l}\right)$ is taken as the estimate ${\bar{D}}_{k}^{*}$, where *n_l_* = *x_thl_*.

### 5.3. Example Illustrating Obtaining ${\bar{V}}_{k}^{*}$ for Small Objects with Uneven Heating

**Figure 17:**
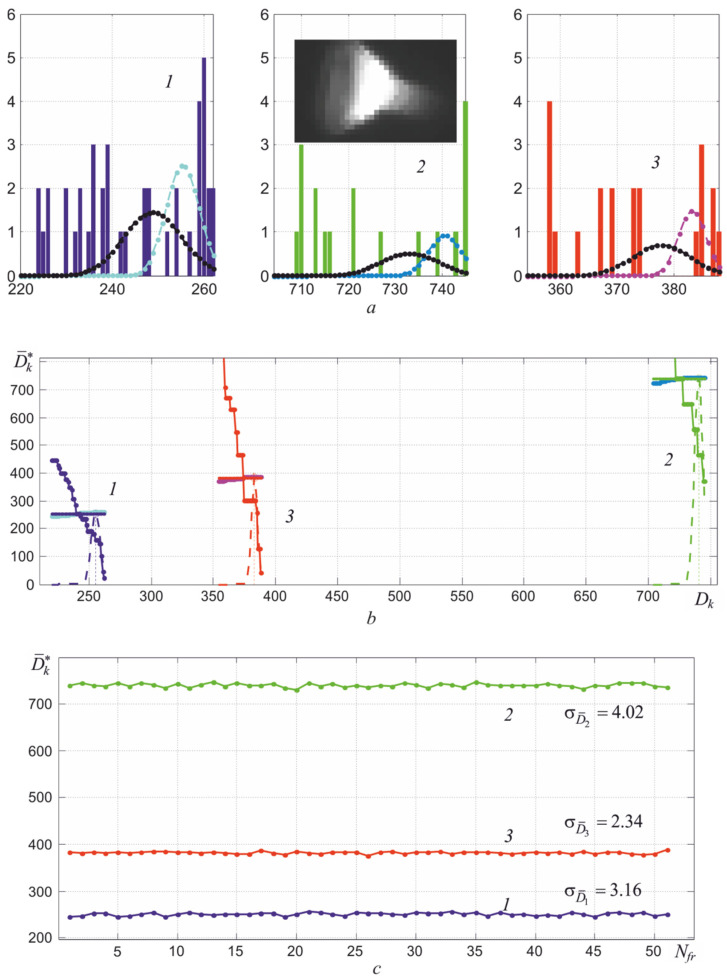
Histograms of counts distribution during registration of heat fluxes generated by a lighting lamp (under conditions *z* = 90 cm and *T_r_*_2_ = 1100 °C) for 1, 2, and 3 spectral regions (**a**) and illustration of the process of determining the average value of digital signals ${\bar{D}}_{k}$ (**b**), as well as the dependence of the resulting estimates ${\bar{D}}_{k}$ on the frame number *N_fr_* (**c**).

Examples of the estimates ${\bar{D}}_{k}^{*}$ obtained in three spectral regions when controlling the temperature of the tungsten filament of a spiral lighting lamp are shown in Figure 17. The thermal image of the spiral of this lighting lamp in the second spectral region when viewed from the side is shown in the inset of Figure 17a. Uneven heating of the surface leads to stretching of the histograms towards lower signal values, which is illustrated by the dependencies in Figure 14d. In contrast to the dependencies shown in Figure 16, with uneven heating, the sum $S_{xW}\left(n\right)$ after crossing the dependence $S_{xW}\left(n\right)/S_{F}\left(n\right)$ continues to increase, as shown in Figure 17b. However, the estimated value ${\bar{D}}_{k}^{*}$ at the point of intersection of the dependencies changes only slightly due to the low steepness of the ratio $S_{xW}\left(n\right)/S_{F}\left(n\right)$. The described method for determining the average value of digital signals ${\bar{V}}_{k}^{*}$ on the right slope of the histogram was specially developed to obtain stable readings of the maximum temperature *T*_max_ when inspecting bodies with uneven heating. Only 18 brightest pixels fall into the averaging interval in the second part of the spectrum. It is clearly seen that the shape of the high-temperature sections of the histograms has a random form with the presence of gaps between their bins or pockets.

The estimation of the current variance can be carried out by calculating the second moment of the distribution *W* (*x*), similarly to calculating the average value. Verification of the effectiveness of using this approach for evaluation $\sigma_{\Sigma Dk}^{}\left(V_{k}\right)$ showed that it leads to underestimated values in comparison with the dependences obtained in the course of calibration, shown in Figure 13. Therefore, the Gaussian approximations of the resulting histograms shown in Figure 17a, with lighter curves with dots, become narrower due to the limited sample, which increases the scatter of the determined values ${\bar{D}}_{k}^{*}$. The use of dependencies in the calculation algorithm predetermined when calibrating the thermograph $\sigma_{\Sigma Dk}^{}\left(V_{k}\right)$ makes it possible to reduce the scatter of the estimate ${\bar{D}}_{k}^{*}$, which is well illustrated in Figure 17c. Figure 17b illustrates the process of automatic search for an estimate ${\bar{D}}_{k}^{*}$ at the intersection of the dependencies $S_{xWk}\left(n\right)/S_{Fk}\left(n\right)$ and $S_{xWk}\left(n\right)$. The root-mean-square deviation $\sigma_{{\bar{D}}_{k}}$ of the obtained estimates is small and ranges from two to four ADC readings, which ensures the stability of the calculated values of the maximum temperature of partial radiation *T_r_*_max_ even with small sizes of the thermal image of an object with uneven heating.

## 6. Correction of the Deviation of the Real Characteristics of the Photodetector Array from the Calculated Ones

As shown above, algorithms for determining the temperature *T* in high-temperature thermography with an uncertainty in the value of the thermal emissivity must contain an option for calculating *T* and ε*_eff_*_2_. It is based on solving a system of three nonlinear Equation (14) with the imposition of additional conditions on the behavior of the spectral coefficient of thermal radiation ε (λ). In the final expressions for calculating *T* and ε*_eff_*_2_, three estimates ${\bar{V}}_{k}^{*}$ of the recorded values are used, averaged over the brightest areas of the thermal image of the object. Usually, in these areas, the thermal conductivity is the best, that is, the surface is the least oxidized. Additionally, two more groups of previously obtained temperature dependences are used. The first of them is the calibration dependences *F*_1_ (*T*) = *F*_RF-GF_ (*T*), *F*_2_ (*T*) = *F*_RF_ (*T*), and *F*_3_ (*T*) = *F*_BF_ (*T*), which are determined during calibration according to the black body model. The second group is the calculated temperature dependences μ_2_(*T*) = μ_RF_ (*T*) and μ_3_(*T*) − μ_1_(*T*) = μ_BF_ (*T*) − μ_RF-GF_ (*T*).

With knowledge of the spectral quantum efficiencies of the photosensitive elements of the matrix η*_k_* (ε) and taking into account the parameters of the calibration scheme, the calculated or hypothetical dependences *F_hk_* (*T*) and the calibration dependences *F_k_* (*T*) determined experimentally during calibration should coincide. However, the inevitable deviations η*_k_* (λ) of the elements of photodetectors matrices, lens parameters, and transmission coefficients τ*_of_* (λ) of optical filters during their manufacture from the averaged characteristics given in the documentation lead to differences in real dependences from those calculated. This is the basis for calibrating thermographs using standard emitters.

**Figure 18:**
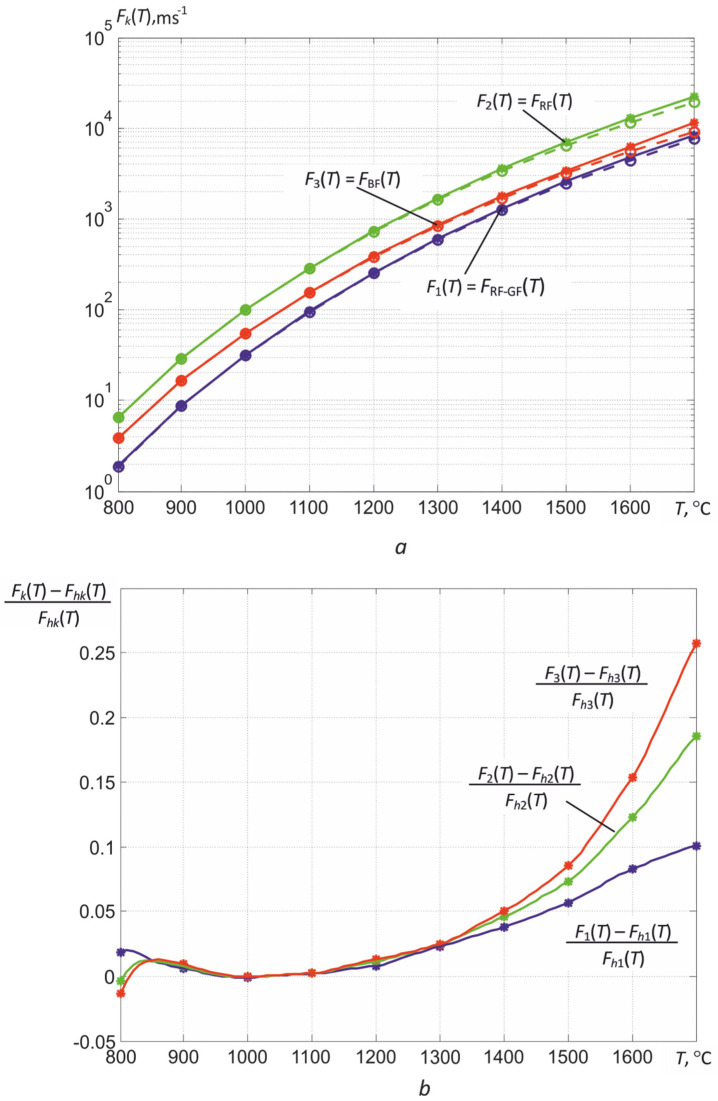
Experimental *F_k_* (*T*) (solid lines) and corrected calculated *F_hk_* (*T*) (dashed lines) calibration curves for a three-spectral thermograph sample based on the MT9V034C12STC matrix (**a**), as well as relative deviations of the experimental dependences from the calculated ones (**b**).

If it is necessary to measure *T* in one wide range of measured temperatures, it is necessary to take into account the deviations of the real characteristics of the photodetector array from the calculated ones. Comparison of the calibration dependences *F_k_* (*T*), obtained during the calibration according to the black body model M390, with the calculated ones *F_hk_* (*T*) shows that the values of *F_k_* (*T*) at high temperatures exceed the calculated values. The dependencies in Figure 18, obtained in the temperature range from 800 to 1700 °C with a discreteness of 100 °C, clearly demonstrate that, at high temperatures, i.e., at low frame exposure times τ*_e_*, a noticeable nonlinearity of the light characteristic of the MT9V034C12STC matrix in the global exposure mode appears. It is difficult to identify the reasons for this nonlinearity, since the technical documentation does not contain information about the used scheme for accumulating and reading signals generated by photodiodes. Only the dynamic range of illumination change is given, which is more than 55 dB in linear mode.

**Figure 19:**
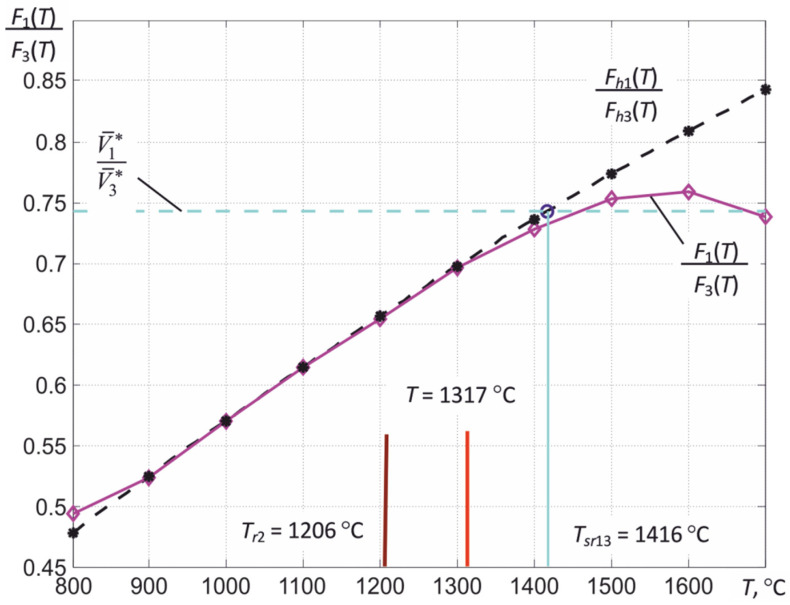
The temperature dependence of the *F*_1_ (*T*)/*F*_3_ (*T*) ratio obtained by calibrating the available thermograph sample (solid line) and the calculated *F_h_*_1_ (*T*)/*F_h_*_3_ (*T*) (dashed line), as well as an illustration of the process of their use in determining the temperature of the spectral ratio *T_sr_*_13_ of a tungsten tape of a standard incandescent lamp SI10–300 at a temperature of partial radiation of its most heated section *T_r_*_2_ = 1206 °C.

This behavior of the calibration curves does not cause problems in determining the temperatures of partial radiation *T_rk_* by comparing the measured values of the rise rates of digital readings ${\bar{V}}_{k}^{*}$ with the calibration curves (see Figure 3). However, when calculating the temperature of the spectral ratio *T_sr_*_13_, the true temperature and the effective coefficient of thermal radiation ε_eff2_, the nonlinearity of the light characteristic can lead to ambiguity of solutions. The foregoing is well illustrated by the temperature dependence of the ratio *F*_1_ (*T*)/*F*_3_ (*T*) for the available sample of the thermograph, shown in Figure 19. If the calculated ratios *F_h_*_1_ (*T*)/*F_h_*_3_ (*T*) are increased monotonically with increasing temperature, then a maximum is observed for the ratios of the obtained calibration curves *F*_1_ (*T*)/*F*_3_ (*T*), after which this ratio decreases. Obviously, starting from some values of *T*, it will not be possible to unambiguously determine the temperature of the spectral ratio *T_sr_*_13_ from the obtained relation. Similarly, corresponding distortions can arise when solving the system of nonlinear equations used to determine *T*.

In the case of nonlinearity of the light characteristic, it is necessary to use the correction of the registered calibration dependences *F_k_* (*T*) and measured values when activating the option for calculating ε_eff2_ and the true temperature *T,* as well as the temperature of the spectral ratio *T_sr_*_13_. This simple correction consists of modifying the obtained discrete calibration dependences *F_k_* (*T_m_*) by multiplying by discrete values of the correcting functions *k_Fk_* (*T_m_*),

(25) $$F_{ck}\left(T_{m}\right)=k_{Fk}\left(T_{m}\right)F_{k}\left(T_{m}\right),$$

the values of which are determined after calibrating the thermograph chamber according to the black body model.

The method for calculating *k_Fk_* (*T_m_*) is as follows. First, the calculated dependences *F_hk_* (*T_m_*) are combined with the experimental ones *F_k_* (*T_m_*) at the calibration point *T*_0_, where the light characteristic of the matrix is almost linear. For example, for the MT9V034C12STC matrix, this point, as follows from Figure 18b, corresponds to the temperature of the radiating core of the black body model 1000 °C. Then, the initial values of the one-dimensional array of the discrete correcting function *k_Fk_*_0_ (*T_m_*) will be equal to the ratio *F_k_* (*T*_0_)/*F_hk_* (*T*_0_). This operation is allowable, since in nonlinear Equations (20) and (23), only the ratio s ${\bar{V}}_{k}^{*}$ (T*)/*F_k_* (T*) are used. The method for calculating *k_F k_*(*T_m_*) is as follows.

Changes in the values of the experimental calibration dependences *F_k_* (*T_m_*) are mainly caused by the inaccuracy of setting the lens f-number, which leads to a deviation of the actual *D/f_l_* ratio from the value used in theoretical calculations. These ratios, even for lenses of the same series, slightly differs from each other, which leads to a parallel shift of the experimental calibration dependences *F_k_* (*T_m_*) up or down due to small differences in the coefficient *K* (3). In some cases, small shifts of the calibration dependences *F_k_* (*T_m_*) relative to each other are observed, which is associated with insignificant displacements of the microlens arrays relative to the photosensitive elements of CMOS matrices at the production stage.

Further, for the calibration points located to the right of *T*_0_, i.e., for *T_m_* > *T*_0_, new values of the correcting function are calculated

(26) $$k_{Fk}\left(T_{m}\right)=\frac{k_{Fk0}\left(T_{m}\right)F_{hk}\left(T_{m}\right)}{F_{k}\left(T_{m}\right)},$$

and for points with *T_m_* < *T*_0_, the old values of *k_Fk_*_0_ (*T_m_*) remain. In this case, we obtain an array of values of the correcting function *k_Fk_*(*T_m_*), for which the first values at *T_m_* ≤ *T*_0_ are equal to *F_k_* (*T*_0_)/*F_hk_* (*T*_0_), and the rest are additionally multiplied by the ratios *F_hk_* (*T_m_*)/*F_k_* (*T_m_*). The corrected calibration curves *F_ck_* (*T_m_*) are close to the calculated ones *F_hk_* (*T_m_*).

Correction of the obtained values ${\bar{V}}_{k}^{*}$ is somewhat more complicated, since it is necessary to take into account the weakening of heat fluxes due to the influence of the thermal radiation coefficient, which for real bodies is less than unity.

When using the simplified method, it is advisable to pre-form the arrays of differences Δ*F_ck_* (*T_m_*) = *F_k_* (*T_m_*) − *F_hk_* (*T_m_*). Then, the correction of the recorded values of ${\bar{V}}_{k}^{*}$ *V*_II*k*_ can be carried out using the spline interpolation log_2_[${\bar{V}}_{k}^{*}$(*T_rk_*)] between the calibration points log_2_[*F_k_*(*T_m_*)] with further subtraction of the value of the interpolated difference Δ*F_ck_* (*T_rk_*) multiplied by the used effective thermal radiation coefficient

(27) $${\bar{V}}_{ck}^{*}\left(T_{rk}\right)={\bar{V}}_{k}^{*}-\varepsilon_{эфk}\Delta F_{ck}\left(T_{rk}\right),$$

where *T_rk_*—calculated from the registered values ${\bar{V}}_{k}^{*}$ of the temperature of partial radiation.

## 7. Discussion

The development of modern technologies for the production of complex products leads to the creation of new methods for their control. These methods and the corresponding equipment should provide the necessary reliability of control and ease of use. Non-contact measurement of high temperatures has features associated with a change in the coefficient of thermal radiation of the monitored objects during their heating and fluctuations of the recorded heat fluxes [8,9,10,24,25]. These fluctuations are caused by changes in the surface temperature of the controlled objects due to the effect of convection air flows, as well as the statistical nature of the emission of photons from the heated surface. Further development of high-temperature thermography requires the introduction of algorithms that allow one to take into account the influence of the unknown thermal radiation coefficient ε and the noted fluctuations of the recorded heat fluxes.

Currently used high-temperature thermographs use monochrome photodetector arrays and registration of thermal radiation in one part of the spectrum. They are used to control processes with consistently repeating parameters. This makes it possible, in the absence of information about the value of the thermal emissivity of the monitored objects, to use only the conditional temperature, i.e., the temperature of partial radiation *T_r_*. In this case, temperature deviations of technological processes are monitored by the deviation of *T_r_* from the specified limits of the range of permissible values.

If it is necessary to measure the temperature close to the true temperature during the adjustment of the thermal process, it is necessary to use methods and thermographic equipment that take into account the influence of the unknown coefficient of thermal radiation and the inevitable presence of fluctuations in the intensity of the recorded fluxes of thermal radiation. The methods used in LWIR thermal imaging to reduce the influence of ε and environmental radiation are not applicable in high temperature thermography. Therefore, researchers from different countries show interest in multispectral thermography and create appropriate laboratory samples [10,11,12,13]. Numerical modeling and practical testing of multispectral thermography methods convince that to solve practical problems of monitoring thermal processes, it is sufficient to use the registration of thermal in three parts of the spectrum. Although in difficult cases, for example, when monitoring laser hardening or welding processes, it is possible to use methods based on processing the spectrum of thermal radiation formed in the area of exposure to a laser beam [26].

In the most demanded temperature range from 800 to 1600 °C, typical for technological processes of heat treatment of ferrous metals, thermographs based on RGB photodetectors matrices can be used to register thermal radiation at the junction of the visible and NIR spectral ranges. The use of an external bandpass filter and a combination of signals generated by an RGB matrix makes it possible to register thermal radiation simultaneously in three overlapping spectral regions. To increase the stability of the solution of a system of three nonlinear equations, it is possible to use a priori information about the value of the slope *b* of the dependence ε (λ) of the clean unoxidized surface of the processed materials, which can be found in reference books. To take into account the oxidation of the surface by atmospheric oxygen when heated to high temperatures, one can use the relationship between the values of *b* and ε, since during oxidation, ε increases, and *b* falls almost to zero.

The existing inhomogeneity of the heating of bodies and the dynamic change in the image of the temperature field formed by the thermograph necessitates the introduction of the option for calculating the maximum temperature *T*_max_ into the algorithms. Moreover, the algorithms for calculating *T*_max_ should ensure the invariance of the determined values of the maximum temperature to the size of the image of the monitored bodies and take into account the variance of fluctuations of the recorded signals.

If it is necessary to form a wide range of measured temperatures, it is necessary to take into account the nonlinearity of the light characteristics of the photodetector matrix and the dependence of the dispersion of its noise on the intensity of the recorded heat fluxes, using the proposed methods for correcting the calibration dependences and recorded signals.

Compliance with the basic principles of reducing the uncertainty of temperature measurement and the introduction of the proposed solutions into the algorithms of operation allows creating, on the basis of modern RGB matrices of silicon photodetectors, rather cheap thermographic equipment for setting up and subsequent control of complex thermal processes.

## Data Availability

Not applicable.
